# *C-reactive protein* genetic variant is associated with diabetic retinopathy in Chinese patients with type 2 diabetes

**DOI:** 10.1186/s12902-015-0006-5

**Published:** 2015-03-02

**Authors:** Danfeng Peng, Jie Wang, Rong Zhang, Shanshan Tang, Feng Jiang, Miao Chen, Jing Yan, Xue Sun, Tao Wang, Shiyun Wang, Yuqian Bao, Cheng Hu, Weiping Jia

**Affiliations:** Shanghai Diabetes Institute, Shanghai Key Laboratory of Diabetes Mellitus, Shanghai Clinical Center for Diabetes, Shanghai Jiao Tong University Affiliated Sixth People’s Hospital, 600 Yishan Road, Shanghai, 200233 China

**Keywords:** C-reactive protein, Diabetic retinopathy, Single nucleotide polymorphism, Inflammation, Type 2 diabetes

## Abstract

**Background:**

Diabetic retinopathy (DR) is an important microvascular complication of diabetes with a high concordance rate in patients with diabetes. Inflammation is supposed to participate in the development of DR. This study aimed to investigate whether genetic variants of *CRP* are associated with DR.

**Methods:**

A total of 1,018 patients with type 2 diabetes were recruited in this study. Of these patients, 618 were diagnosed with DR, 400 were patients with diabetes for over 10 years but without DR, considered as cases and controls for DR, respectively. Four tagging SNPs (rs2808629, rs3093077, rs1130864 and rs2808634) within *CRP* region were genotyped for all the participants. Fundus photography was performed for diagnosis and classification for DR.

**Results:**

rs2808629 was significantly associated with increased susceptibility to DR (odds ratio 1.296, 95% CI 1.076-1.561, *P* = 0.006, empirical *P* = 0.029, for G allele). This association remained significant after adjustment for confounding factors (odds ratio 1.261, 95% CI 1.022-1.555, *P* = 0.030).

**Conclusions:**

In this study, we found *CRP* rs2808629 was associated with DR in the Chinese patients with type 2 diabetes.

## Background

Diabetes mellitus, especially type 2 diabetes mellitus (T2DM), has become a global epidemic. With the rapid rising incidence of T2DM, diabetic complications will certainly pose a major public health concern in the coming decades. Diabetic retinopathy (DR) is a major microvascular complication of diabetes and ranks as the leading cause of blindness among working-aged adults around the world [1]. Epidemiological studies have shown that the incidence of DR parallels with diabetes duration and intensive glycaemic control can delay its development [2]. However it is noteworthy that some patients with good glycaemic control may still develop DR whereas some are spared from DR despite poor glycaemic control. Although the mechanisms underlie this observation remains largely unknown, inherited susceptibility may modulate the risk of DR because retinopathy aggregates in families. Siblings of patients with T2DM who have DR showed a significant higher risk of DR compared to siblings of patients with T2DM who have no DR [3,4]. Significant familial influence on the severity of DR was also observed [5,6] and the risk of DR varies among different ethnic groups [7-9]. To data, although no locus for DR from genome-wide association study (GWAS) has reached conventional significance criteria, but a significant number of genes and genetic variants have been proposed for DR or proliferative DR through candidate gene approach [10,11]. Several pathways and processes, including the renin-angiotensin system, vascular endothelial dysfunction, tissue matrix remodeling, and angiogenesis, have been strongly implicated in the pathogenesis of DR, and multiple genes involved in these pathways have been identified for DR (e.g., *AKR1B1*, *VEGFA*, *ACE*, and *AGER*).

C-reactive protein (CRP), a very sensitive marker of inflammation produced by the liver cells in response to various stimuli, is involved in endothelial dysfunction and angiogenesis [12,13] which have been proposed to play an important role in the pathogenesis of DR [14,15]. It is now well accepted that CRP is a strong predictor of future cardiovascular events [16,17]. However, less is known about its relationship with microvascular complications of diabetes. In this regard, some, though not all, studies (especially prospective studies) have reported that circulating CRP is associated with diabetic nephropathy (DN) [18,19] and DR [15,20] in the last decade, raising the possibility that the increase of CRP may be an early event or even one of driving forces in the development of microvascular complications of diabetes. Therefore, it is plausible to hypothesize that genetic variants of *CRP* may have impact on the risk of these complications. In the current study, we investigated the association between *CRP* variants and DR in a Chinese population.

## Methods

### Participants

This study involved 1,018 patients with T2DM recruited from the Shanghai Diabetes Institute Inpatient Database of Shanghai Jiao Tong University Affiliated Sixth People’s Hospital. All participants were unrelated patients with T2DM meeting the 1999 WHO criteria (fasting plasma glucose ≥ 7.0 mmol/l and/or 2 h plasma glucose ≥ 11.1 mmol/l). Type 1 diabetes and mitochondrial diabetes were excluded by clinical, immunological (individuals with GAD and/or protein tyrosine phosphatase IA-2 antibodies were excluded) and genetic methods (mitochondrial tRNA^Leu(UUR)^ A3243G mutation carriers were excluded). Of these patients, 618 were diagnosed with DR, 400 were patients without DR, considered as cases and controls for DR, respectively. For controls selection, patients with diabetes for over 10 years were chosen deliberately. This study was approved by the institutional review board of Shanghai Jiao Tong University Affiliated Sixth People’s Hospital, with written informed consent obtained from each participant.

### Clinical measurement

Each participant completed a standard questionnaire for detailed information as described previously [21]. Fundus photography was performed according to a standardized protocol at the Department of Ophthalmology, Shanghai Jiao Tong University Affiliated Sixth People’s Hospital. Both eyes of each patient were photographed with a 45-degree 6.3-megapixel digital nonmydriatic camera (Canon CR6-45NM, Lake Success, NY). A five-stage disease severity classification for DR was applied according to the International Classification of Diabetic Retinopathy [22]: no apparent retinopathy (no abnormalities), mild nonproliferative diabetic retinopathy (NPDR) (microaneurysms only), moderate NPDR (more than just microaneurysms but less than severe NPDR), severe NPDR (more than 20 intraretinal hemorrhages in each of 4 quadrants and/or definite venous beading in 2 quadrants and/or prominent intraretinal microvascular abnormalities in 1quadrant and no signs of proliferative retinopathy), or proliferative diabetic retinopathy (PDR) (neovascularization and/or vitreous hemorrhage and/or preretinal hemorrhage). DR grade was evaluated for both eyes, and higher grade was recorded for each person. Of the 618 patients with DR in this study, there were 395 with mild NPDR, 103 with moderate NPDR, 84 with severe NPDR, and 36 with PDR. Body mass index (BMI) was calculated as weight in kilograms divided by height in meters squared. Glycaemic control was evaluated by measuring glycated haemoglobin (HbA1c) levels. Data of blood pressures and lipid profiles were also collected for each participant. Hypertension was defined as systolic blood pressure ≥ 140 mmHg and/or diastolic blood pressure ≥ 90 mmHg.

### Single nucleotide polymorphisms (SNPs) selection, genotyping and quality control

In this study, we selected four tagging SNPs (rs2808629, rs3093077, rs1130864 and rs2808634) that spanned 11 kb in the upstream and 6 kb in the downstream region of *CRP*, according to HapMap phase III (release 28) Han Chinese database with a threshold of r^2^ ≥ 0.8. We could tag 95% SNPs (21 out of 22 SNPs) with a minor allele frequency (MAF) > 0.01 within *CRP* region. All genotyping was done using the primer extension of multiplex products with detecting by matrix-assisted laser desorption ionization-time of flight mass spectroscopy using a MassARRAY Compact Analyzer (Sequenom, San Diego, CA, USA). The genotyping data underwent a series of quality control checks and cleaned data were used in further statistical analysis. The call rates for rs2808629, rs3093077, rs1130864 and rs2808634 were 97.0%, 95.9%, 98.3% and 96.9%, respectively. The concordance rates based on 100 duplicates were over 99% for all these SNPs. Thirty-seven individuals were excluded from the sample call rate checks. The Hardy-Weinberg equilibrium test was performed before the association analysis, and all the four SNPs were in accordance with Hardy-Weinberg equilibrium (*P* = 0.68 for rs2808629, *P* = 0.74 for rs3093077, *P* = 0.34 for rs1130864 and *P* = 0.47 for rs2808634, respectively).

### Statistical analysis

The allelic frequencies between the patients with or without DR were compared by *χ*^2^ test, and odds ratios with 95% confidence intervals (CIs) were presented. Genotype distributions between patients with or without DR were compared using multiple logistic regressions under an additive model with adjustment for confounding factors. The effects of SNPs on the levels of retinopathy severity were analyzed by trend analysis. All these analyses were performed using SAS 9.3 (SAS institute, Cary, NC, USA) unless specified otherwise. A two-tailed *P* value < 0.05 was considered statistically significant.

On the basis of the previously reported effect size of genetic loci for DR (~1.40) [10], our samples had > 90% power to detect an effect SNP with MAF of 0.3 and > 80% power to detect an effect SNP with MAF of 0.2 at a level of significance of 0.05.

## Results

The clinical characteristics of the subjects passed genotype quality control were shown in Table 1. Compared with patients without DR, patients with DR were diagnosed with diabetes at earlier age and had higher HbA1c levels and higher prevalence of hypertension and diabetic nephropathy. Besides, as patients without DR with diabetes for over 10 years were selected in our study, they were older and had longer duration of diabetes compared with patients with DR.

**Table 1:** **Clinical characteristics of the study patients**

**Characteristic**	**Diabetic retinopathy**	**Patients without retinopathy**	***P*** **value**
	**(n = 593)**	**(n = 388)**	
Male/female (n)	285/308	154/234	0.010
Age (years)	62.48 ± 10.79	67.37 ± 9.62	<0.0001
BMI (kg/m^2^)	24.12 ± 3.65	24.02 ± 3.36	0.861
Age at diagnosis of diabetes (years)	51.70 ± 10.60	53.39 ± 9.87	0.014
Duration of diabetes (years)	10.00(5.00,15.00)	12.00(10.00,16.00)	<0.0001
HbA1c (%)	9.16 ± 2. 17	8.70 ± 2.11	0.0003
Systolic blood pressure (mmHg)	138.66 ± 18.90	135.98 ± 17.83	0.014
Diastolic blood pressure (mmHg)	81.39 ± 9.55	79.34 ± 9.45	0.001
Subjects with hypertension [n (%)]	335(56.5%)	186(47.9%)	0.009
Subjects with nephropathy [n (%)]	232(39.1%)	113(29.1%)	0.001

Data are n, mean ± SD or median (interquartile range). BMI, body mass index.

25 patients with DR and 12 patients without DR were excluded due to sample call rate check.

We firstly analyzed the association between these SNPs and the risk of DR. As shown in Table 2, rs2808629 was nominally associated with increased susceptibility to DR (odds ratio 1.296, 95% CI 1.076-1.561, *P* = 0.006, for G allele). This association remained significant after adjusting for confounding factors, including HbA1c levels, duration of diabetes, systolic and diastolic blood pressure, BMI and sex (odds ratio 1.261, 95% CI 1.022-1.555, *P* = 0.030), as well as adjusting for multiple comparisons (empirical *P* = 0.029). The other SNPs did not show any association with DR. Further, we tried to examine the effect of rs2808629 on the disease severity of DR. Of the 593 DR patients, there were 379 patients with mild NPDR, 98 with moderate NPDR, 80 with severe NPDR and 36 with PDR. However, no significant association of rs2808629 with DR severity was observed (*P* = 0.387 for trend analysis). The distributions of these four SNPs among patients with different levels of DR were shown in Table 3. In addition, rs2808629 was genotyped among 438 subjects with normal glucose regulation. The distribution of this SNP was similar to those reported in other studies of Asian populations in the HapMap database (G allele frequency 0.453 vs 0.419/0.297/0.537), indicating that there was no technical error in this study.

**Table 2:** **Associations of** ***CRP*** **SNPs with diabetic retinopathy**

**SNP**	**Chr: position (Build 38)**	Reference position*	**Major/minor allele**	**Risk allele**	**Cases**		**Controls**		**OR (95% CI)**	***P*** **value (empirical** ***P*** **value)**	**OR (95% CI)** ^**§**^	***P*** **value** ^**§**^
					**(n = 593)**		**(n = 388)**					
					**Minor allele frequencies**	**Genotype count 11/12/22** ^**#**^	**Minor allele frequencies**	**Genotype count 11/12/22** ^**#**^				
rs2808629	1:159707006	+5283	A/G	G	0.452	171/297/115	0.389	141/180/57	1.296(1.076,1.561)	**0.006**(**0.029**)	1.261(1.022,1.555)	**0.030**
rs3093077	1:159709846	+2443	T/G	G	0.183	390/179/18	0.163	271/106/10	1.153(0.906,1.468)	0.248(0.612)	1.194(0.906,1.574)	0.209
rs1130864	1:159713301	3’ UTR	C/T	T	0.058	520/62/3	0.045	352/33/1	1.299(0.855,1.974)	0.219(0.667)	1.250(0.794,1.968)	0.335
rs2808634	1:159722783	−8194	C/T	T	0.160	420/155/17	0.151	281/97/10	1.070(0.833,1.375)	0.597(0.968)	1.056(0.804,1.386)	0.697

*P* values < 0.05 are shown in bold.

The additive model was used in the association analysis between genotype and DR.

*position of SNP with reference to *CRP*.

^#^11, major allele homozygotes; 12, heterozygotes; 2 2, minor allele homozygotes.

^§^adjusted for duration of diabetes, HbA1c, systolic blood pressure, diastolic blood pressure, body mass index and sex.

Empirical *P* values are based on 10,000 permutations.

The OR with 95% CI shown is for the minor allele.

**Table 3:** ***CRP*** **SNPs distributions among patients with different severities of DR**

**SNP**	**Mild NPDR**		**Moderate NPDR**		**Severe NPDR**		**PDR**	
	**(n = 379)**		**(n = 98)**		**(n = 80)**		**(n = 36)**	
	**Minor allele frequencies**	**Genotype count 11/12/22** ^**#**^	**Minor allele frequencies**	**Genotype count 11/12/22** ^**#**^	**Minor allele frequencies**	**Genotype count 11/12/22** ^**#**^	**Minor allele frequencies**	**Genotype count 11/12/22** ^**#**^
rs2808629	0.465	110/180/84	0.463	25/52/18	0.405	25/44/10	0.386	11/21/3
rs3093077	0.196	242/122/13	0.168	67/24/4	0.163	55/24/1	0.129	26/9/0
rs1130864	0.053	339/34/3	0.072	83/14/0	0.058	69/9/0	0.074	29/5/0
rs2808634	0.166	263/106/10	0.168	69/25/4	0.127	61/16/2	0.139	27/8/1

^#^11, major allele homozygotes; 12, heterozygotes; 22, minor allele homozygotes.

## Discussion

In the present study, we investigated the association of *CRP* variants with DR in Chinese patients with T2DM. It’s the first time to our knowledge to investigate the association between *CRP* variants with DR. Our results revealed that rs2808629 was significantly associated with the risk of DR in Chinese patients with T2DM. This association remained significant after adjusting for multiple comparisons. Moreover, the correlation of rs2808629 with DR stayed significant after adjusting for confounding factors, including duration of diabetes, HbA1c, blood pressure, BMI and sex, implying that this SNP is an independent genetic factor for susceptibility to DR. It has been reported in several studies that *CRP* genetic variants were associated with serum CRP levels [23-28]. rs2808629 was identified to be associated with serum CRP levels in a previous genome-wide association study [23]. Therefore, it is plausible that the effect of rs2808629 on susceptibility to DR is because of its influence on serum CRP levels. However, further studies are needed for confirmation.

As an acute phase reactant, CRP production increases in response to a variety of systemic events such as infection, trauma, or autoimmune inflammatory diseases. Among other systemic inflammatory mediators, CRP has been widely accepted as a potent risk indicator, independently predicting future cardiovascular events in the last decades [29-31]. On the other hand, given the increasingly recognized link between chronic inflammation and microvascular complications [15,32], the relationship between CRP and DR has been investigated in some studies. However, results from limited studies on possible association of CRP with DR are inconsistent. In the Hoorn study [15], a large population-based cohort study of 625 adults, higher CRP was associated with the prevalence of any DR. Another prospective study of inflammatory biomarkers and risk of DR in the Diabetes Control and Complications Trial also indicated that after adjusting for known risk factors, increasing quintiles of baseline high-sensitivity CRP (hsCRP) level may be associated with higher risks of incident clinically significant macular edema and the development of macular hard exudates [33]. However, report from the Wisconsin Epidemiologic Study of Diabetic Retinopathy (WESDR) [34], a longitudinal population based study of persons with type 1 diabetes, did not find any association between CRP and DR. Similar results were reported by the Multi-ethnic Study of Atherosclerosis (MESA) [35]. Besides, Lim et al. [36] reported that patients with higher levels of CRP were less likely to have DR in the Singapore Malay Eye Study (SiMES), a cross-sectional study on 718 persons with diabetes. Nevertheless, emerging evidence supports CRP as an active participant instead of a mere bystander in the pathogenesis of DR. CRP could inhibit endothelium-dependent nitric oxide-mediated dilation in retinal arterioles, thus potentially facilitating the development of retinal vascular diseases [37]. Besides, CRP could stimulate leucocyte-endothelium interactions [38], decrease endothelial nitric oxide [39], and impair the number and function of endothelial progenitor cells [40], thereby promoting endothelial dysfunction, which is another important mediator in the development of diabetic microvascular complications [15]. Taken together, these findings imply that genetic variants of *CRP* may exert significant effects on DR. And our study found a common variant of *CRP*, rs2808629, was significantly associated with DR in the Chinese patients with T2DM.

Some limitations should be noted in our study. Firstly, although the association of rs2808629 with DR remained significant after adjusting for multiple comparisons (empirical *P* = 0.029), we still cannot fully exclude the possibility that the association detected was a false positive. But considering the power of our study samples and the effect of this SNP, the possibility of a false positive is limited. Secondly, rs2808629 locates in the downstream of *CRP*, and we suppose that it may participate in the susceptibility to DR through its effects on regulating CRP expression or it may be just a genetic marker in linkage disequilibrium with the causal variant(s). However, further studies are needed to reveal the underlying mechanism. Thirdly, although we found association of *CRP* variant with DR in Chinese patients with T2DM, whether this effect is restricted to T2DM is still unknown and needs to be investigated in studies among patients with type 1 diabetes and other ethnic groups.

## Conclusions

In summary, we found that *CRP* rs2808629 was associated with DR in Chinese patients with T2DM. This study discover the association of *CRP* variant with DR for the first time, although the mechanism underlying the genotype–phenotype association is unknown, it provides a hypothesis for future researches. Further studies are needed to replicate this finding in other populations and translate the common variant association signal into biological mechanisms of disease causation.

## Abbreviations

T2DM: Type 2 diabetes mellitus
DR: Diabetic retinopathy
DN: Diabetic nephropathy
GWAS: Genome-wide association study
CRP: C-reactive protein
NPDR: Nonproliferative diabetic retinopathy
PDR: Proliferative diabetic retinopathy
HbA1c: Glycated haemoglobin
SNP: Single nucleotide polymorphism
BMI: Body mass index
hsCRP: High-sensitivity CRP
